# Cyanidin-3-O-Glucoside-Rich Black Rice Fraction Attenuates IL-1β/IL-6-Driven A549 Lung Cancer Cell Migration and Invasion and Modulates JAK1/STAT3 Signaling

**DOI:** 10.3390/nu18081198

**Published:** 2026-04-10

**Authors:** Warathit Semmarath, Punnida Arjsri, Kamonwan Srisawad, Intranee Intanil, Sansanee Jamjod, Chanakan Prom-u-thai, Pornngarm Dejkriengkraikul

**Affiliations:** 1 Akkhraratchakumari Veterinary College, Walailak University, Nakhon Si Thammarat 80160, Thailand; 2 Centre for One Health, Walailak University, Nakhon Si Thammarat 80160, Thailand; 3 Department of Biochemistry, Faculty of Medicine, Chiang Mai University, Chiang Mai 50200, Thailand; punnida.dream@gmail.com (P.A.); kamonwan.sri@cmu.ac.th (K.S.); intranee.in@cmu.ac.th (I.I.); 4 Anticarcinogenesis and Apoptosis Research Cluster, Faculty of Medicine, Chiang Mai University, Chiang Mai 50200, Thailand; 5 Department of Plant and Soil Sciences, Faculty of Agriculture, Chiang Mai University, Chiang Mai 50200, Thailand; sansanee.cm@gmail.com (S.J.); chanakan.p@cmu.ac.th (C.P.-u.-t.); 6 Lanna Rice Research Center, Chiang Mai University, Chiang Mai 50200, Thailand

**Keywords:** pigmented black rice, cyanidin-3-O-glucoside, anthocyanins, functional food, tumor microenvironment, inflammation-associated metastasis, IL-6, JAK/STAT signaling, A549 lung adenocarcinoma

## Abstract

Background/Objectives: Inflammatory mediators within the tumor microenvironment contribute to lung cancer progression by enhancing cellular motility and invasive capacity through cytokine-dependent signaling networks. Modulation of these inflammation-associated pathways by dietary bioactive compounds may provide complementary strategies for limiting cancer aggressiveness. Our objective was to examine the inhibitory effects of a cyanidin-3-O-glucoside (C3G)-rich fraction from Kum Akha pigmented black rice (CKAB-P1) on inflammation-stimulated A549 cancer cell progression. Methods: CKAB-P1 was obtained through solvent-partition extraction and chemically characterized using the pH differential method and high-performance liquid chromatography. A549 cells were pretreated with CKAB-P1 or C3G, followed by stimulation with conditioned medium predominantly containing IL-6 and IL-1β derived from LPS-exposed THP-1 macrophages (THP-1-CS). Effects on cancer cell migration and invasion were evaluated using wound-healing, Transwell invasion, gelatin zymography, and Western blot analyses. Results: CKAB-P1 contained 106.62 ± 3.54 mg/g extract of total anthocyanins, with C3G representing the major constituent (59.42 ± 2.54 mg/g extract). Exposure of THP-1-CS stimulated migration and invasion of A549 lung cancer, and neutralization of IL-6 and IL-1β reduced these pro-migratory effects, confirming cytokine involvement. Treatment with CKAB-P1 (10–40 μg/mL) or C3G (2.5–20 μg/mL) markedly attenuated inflammation-enhanced migration and invasion (*p* < 0.05). A reduction in MMP-2 and MMP-9 activity, along with decreased expression of invasion-associated protein expressions (uPA, uPAR, and MT1-MMP), was observed. Furthermore, both CKAB-P1 and C3G attenuated phosphorylation of JAK1 and STAT3. Conclusions: These findings suggest that anthocyanin-enriched black rice fraction may limit inflammation-driven A549 lung cancer cell aggressiveness through modulation of the cytokine-driven JAK1/STAT3 signaling cascade, indicating its potential relevance as a bioactive dietary component targeting tumor-associated inflammatory signaling.

## 1. Introduction

Inflammation exerts a dual role in cancer biology. While effective immune surveillance can eliminate transformed cells, persistent activation of alternative immune populations may instead promote tumor progression. Within the tumor microenvironment, inflammatory cells, particularly macrophages, secrete cytokines and growth factors that enhance malignant cell survival, migration, angiogenesis, and immune evasion [1,2]. A defining feature of such inflammation is its ability to activate abnormality-sensing pathways, including Toll-like receptor (TLR) signaling, through the secretion of inflammatory mediators. This process drives immunosuppressive innate and adaptive immune infiltration and is instrumental across multiple cancer types, including lung cancer [3,4]. Sustained inflammatory signaling therefore contributes directly to tumorigenesis and metastatic dissemination, the principal cause of cancer-related mortality [5].

Lung cancer remains a major global health burden and continues to be the leading cause of cancer-related mortality worldwide. According to the lung tumor classification by the World Health Organization (WHO), non-small cell lung cancer (NSCLC) accounts for approximately 85% of all lung cancer diagnoses and comprises several histological forms, including adenocarcinoma, squamous cell carcinoma, and large cell carcinoma [6]. Despite advances in diagnostic techniques and therapeutic strategies, the prognosis of NSCLC remains poor due to its aggressive growth, early metastatic spread, and frequent development of therapeutic resistance, particularly in patients diagnosed at advanced stages [7,8]. Chronic exposure to carcinogens such as cigarette smoke, asbestos, and silica induces persistent pulmonary inflammation that facilitates malignant transformation and disease progression [9].

Tumor-promoting inflammation may arise from tumor-induced necrosis of surrounding healthy cells. This has led to the release of cell contents and the triggering of pro-inflammatory mediator secretions. In the lung cancer microenvironment, tumor-associated macrophages (TAMs) are key mediators of the inflammatory microenvironment. Among their secreted factors, interleukins majorly contribute to promoting tumor aggressiveness. These cytokines (IL-6, IL-8, IL-1β, and IL-18) activate intracellular signaling pathways, including nuclear factor-κB (NF-κB) and the Janus kinase/signal transducer and activator of transcription (JAK-STAT) cascade [10,11]. Persistent activation of JAK1 and STAT3 has been widely implicated in enhancing the transcription of metastasis-associated genes, including matrix metalloproteinases (*MMP-2* and *MMP-9*), urokinase-type plasminogen activator (*uPA*), and its receptor (*uPAR*), thereby facilitating extracellular matrix remodeling and tumor invasion across multiple cancer types [12,13]. Because inflammation within the tumor microenvironment promotes tumor cell migration and metastasis, targeting inflammation-driven JAK/STAT signaling has emerged as a rational strategy to suppress lung cancer progression.

Black rice (*Oryza sativa* L.), a pigmented rice widely consumed in Asia, has gained attention as a functional food due to its high content of bioactive phytochemicals. The dark-purple-to-black coloration of the grain is attributed to the accumulation of anthocyanins, predominantly localized in the bran layer. In addition to anthocyanins, black rice contains a diverse range of phenolic compounds, including flavonoids, phenolic acids, and proanthocyanidins. Among these, cyanidin-3-O-glucoside (C3G) and peonidin-3-O-glucoside (P3G) are the major anthocyanins and are considered key contributors to the biological activities of black rice.

Polyphenols, including anthocyanins, exert antioxidant activity through multiple mechanisms, such as direct scavenging of reactive oxygen species (ROS), metal ion chelation, and modulation of endogenous antioxidant defense systems [14]. Importantly, oxidative stress and chronic inflammation are closely interconnected processes in cancer progression, and the antioxidant properties of polyphenols are often linked to their anti-inflammatory and anticancer effects [15]. In particular, C3G has been shown to modulate redox-sensitive signaling pathways and suppress pro-inflammatory cytokine production. It should also be noted that polyphenols may exhibit both antioxidant and pro-oxidant activities depending on concentration and cellular context, a phenomenon commonly observed for natural antioxidants [16]. A schematic figure illustrating the chemical structures of major phenolic compounds, including cyanidin-3-O-glucoside (C3G), peonidin-3-O-glucoside (P3G), and representative compounds from major phenolic subclasses (e.g., catechin and gallic acid), is provided in Figure S1.

Bioactive dietary compounds have attracted increasing interest for their ability to modulate inflammatory and oncogenic signaling networks. Pigmented rice varieties are particularly rich in anthocyanins, with cyanidin-3-O-glucoside (C3G) and peonidin-3-O-glucoside (P3G) as the predominant compounds localized in the germ and bran layers [17,18]. Among these, C3G is typically the most abundant anthocyanin and has been extensively studied for its biological activities. Anthocyanins have been widely reported to exhibit antioxidant and anti-inflammatory activities across diverse experimental models [19,20,21,22]. Previous investigations, including our own work, revealed that anthocyanin-rich fractions of pigmented black rice attenuate experimental respiratory inflammation [20]. However, despite these protective effects in inflammatory respiratory settings, the role of anthocyanin-rich black rice fractions in modulating inflammation-enhanced tumor progression remains incompletely defined. Specifically, whether dietary anthocyanins can interrupt macrophage-derived IL-1β/IL-6-mediated activation of the JAK1/STAT3 pathway and consequently suppress metastatic phenotypes in A549 lung cancer cells has not been systematically investigated. Addressing this question is essential for understanding the potential of functional dietary components to modulate pro-metastatic signaling within the tumor microenvironment.

Therefore, this study aimed to investigate the inhibitory effects of the C3G-rich fraction from Kum Akha pigmented black rice germ and bran (CKAB-P1) on inflammation-driven A549 lung cancer cell progression. An in vitro tumor-promoting inflammatory microenvironment was established using conditioned medium from lipopolysaccharide-stimulated THP-1 macrophages enriched in IL-1β and IL-6. The effects of CKAB-P1 and its major anthocyanin, C3G, on migration, invasion, and metastasis-related protein expression in A549 lung cells were evaluated, with emphasis on modulation of the IL-1β/IL-6/JAK1/STAT3 signaling axis.

## 2. Materials and Methods

### 2.1. Rice Materials and Formulation of C3G-Rich Fraction

Kum Akha 1 CMU glutinous black rice variety was obtained from the Lanna Rice Research Center, Chiang Mai University, Thailand. The C3G-rich fraction was prepared using solvent-partition extraction with minor modifications from the established protocol [21]. Briefly, the outer-layer part (germ and bran) was soaked with 50% *v*/*v* ethanol (RCL Labscan Limited, Bangkok, Thailand) for 48 h at 20–25 °C. The samples were then filtered with Whatman^TM^ No. 1 (Cytiva, Wilmington, DE, USA) and evaporated using a rotary evaporator (BUCHI, Flawil, Switzerland). The subsequent extract was separated with saturated *n*-butanol (RCL Labscan Limited, Bangkok, Thailand) by a separatory funnel. The upper part with medium polarity is designated as the butanol fraction and was named CKAB-P1. While the remaining lower part, designated the aqueous fraction (CKAB-P2), was collected separately. Solvents were removed by evaporation, and the fractions were freeze-dried and stored until further analysis.

### 2.2. Phytochemical Analysis

Total phenolic content (TPC) was assessed by spectrophotometry using the Folin–Ciocalteu method with minor modifications from the previously described protocol [23]. The results were expressed as milligrams of gallic acid equivalents per gram of extract (mg GAE/g extract). Total flavonoid content (TFC) was assessed by spectrophotometry (Tecan, Männedorf, Switzerland) using the aluminum chloride colorimetric assay, following the previously described protocol [24]. The results were expressed as milligrams of catechin equivalents per gram of extract (mg CE/g extract). Total anthocyanin content (TAC) was determined by spectrophotometry using the pH differential method with minor modifications from the previously described protocol [25,26]. Total anthocyanin content was calculated using the equation formula based on C3G concentration (mg C3G/g extract), which has been described elsewhere [25].

### 2.3. HPLC Analysis of Anthocyanins

The protocol for anthocyanin detection was modified from a previously described method [27]. Reversed-phase HPLC using an acidified mobile-phase system was employed to enhance the stability of anthocyanins and to improve peak resolution and reproducibility. The anthocyanin composition of CKAB fractions was analyzed using an Agilent 1260 Infinity HPLC system (Agilent Technologies, Santa Clara, CA, USA). Separation was performed on a Zorbax Eclipse Plus C18 column (250 mm × 4.6 mm, 5 µm; Agilent Technologies) using reversed-phase chromatography. The mobile phase consisted of 0.4% trifluoroacetic acid (RCL Labscan Limited, Bangkok, Thailand) in water as solvent A and 0.45% trifluoroacetic acid in acetonitrile (RCL Labscan Limited, Bangkok, Thailand) as solvent B under isocratic conditions. The signal was detected at 520 nm. Anthocyanin concentrations were quantified by comparing peak areas with commercially available C3G and P3G standards (Sigma-Aldrich, St. Louis, MO, USA) and expressed as mg/g extract.

### 2.4. Determination of Antioxidant Activity

The in vitro antioxidant activities of CKAB fractions were assessed using ABTS and DPPH radical-scavenging assays with minor modifications from established protocols [28,29]. For the ABTS assay, the spectrophotometry signal was detected at 734 nm and compared with a Trolox calibration curve. For the DPPH assay, the spectrophotometry signal was detected at 540 nm and compared with a vitamin E standard curve. Radical-scavenging activity was expressed as the half-maximal inhibitory concentration (IC_50_, μg/mL).

### 2.5. Cell Line and Culture

The lung adenocarcinoma A549 cells (American Type Culture Collection: ATCC) were cultured in DMEM (Gibco BRL, Grand Island, NY, USA). supplemented with 10% fetal bovine serum (FBS), 2 mM L-glutamine, 50 U/mL penicillin, and 50 μg/mL streptomycin at 37 °C in a humidified atmosphere containing 5% CO_2_.

The THP-1 cells (ATCC, TIB-202™) were cultured in RPMI-1640 medium (Sigma-Aldrich, St. Louis, MO, USA) supplemented with the same conditions as A549 cells. Macrophage differentiation followed the previously described protocol [30]. THP-1 cells were induced with 0.01 μg/mL of PMA (Sigma-Aldrich, St. Louis, MO, USA) for 24 h. Following differentiation, cells were maintained in PMA-containing medium for an additional 24 h, then washed and cultured in PMA-free RPMI medium for 24 h to allow resting prior to experimental stimulation.

### 2.6. Cytotoxicity Assay

The cell viability of A549 cells was assessed using the MTT assay following the previously described protocol [22]. The cells were seeded at 3 × 10^3^ cells per well in 96-well plates and treated with CKAB-P1 (0–200 μg/mL) or C3G (0–20 μg/mL) for 24 or 48 h. Following treatment, MTT dye (Sigma-Aldrich, St. Louis, MO, USA) was added and incubated for an additional 4 h. The results were represented as percentage cell viability relative to untreated controls.

### 2.7. Determination of Cytokine Secretion

To generate inflammation-conditioned medium, differentiated THP-1 macrophages (6.5 × 10^5^ cells/well in 6-well plates) were stimulated with 1 μg/mL LPS (Sigma-Aldrich, St. Louis, MO, USA) for 24 h. PMA-differentiated THP-1 macrophages followed by LPS stimulation are widely reported to exhibit a pro-inflammatory (M1-like) phenotype, characterized by increased secretion of cytokines such as IL-6 and IL-1β [31,32]. Culture supernatants were collected and centrifuged to remove debris. Cytokine levels (IL-6, IL-1β, IL-8, and IL-18) were quantified using ELISA kits according to the manufacturer’s instructions (BioLegend, San Diego, CA, USA). Absorbance was measured at 450 nm with reference to 570 nm using a microplate reader (Tecan, Männedorf, Switzerland). The resulting supernatant was designated THP-1-conditioned medium (THP-1-CS) and used to establish the tumor-promoting inflammatory-microenvironment model.

### 2.8. Scratch Assay for Detection of Cancer Cell Migration

The scratch assay was used to determine the effects of inflammatory stimulation and the inhibitory activity of CKAB-P1 and C3G on A549 cell motility. A549 cells were seeded at 2.5 × 10^5^ cells per well in 6-well plates and cultured for 24 h. A sterile 200 μL pipette tip was used to create a linear wound across the cell. To assess the pro-migratory effect of inflammatory stimulation, cells were exposed to standardized THP-1-conditioned medium (THP-1-CS), enriched in IL-6 (234.88 ± 7.36 pg/mL) and IL-1β (124.55 ± 10.25 pg/mL) with a volume of 2 mL, for 24 h. For cytokine neutralization experiments, anti-IL-6 and anti-IL-1β antibodies (1:1000) were added to evaluate the specific contribution of these cytokines to A549 cell migration. To evaluate the inhibitory effects of CKAB-P1 and C3G, A549 cells were pretreated with CKAB-P1 or C3G for 4 h prior to stimulation with THP-1-CS. To minimize potential proliferation-related confounding effects, the cells were treated and incubated in DMEM containing 0.5% FBS under inflammatory conditions for an additional 24 h. Images of the same wound areas were captured at 0 and 24 h using a phase-contrast microscope (Nikon Eclipse TS100, Nikon Instruments Inc., Tokyo, Japan) at ×100 magnification. Wound area was quantified using the “Wound Healing Size Tool” plugin in ImageJ software (version 1.410) as previously described [33]. Plugin parameters were applied consistently across all groups to ensure accurate delineation of wound boundaries.

### 2.9. Transwell Assay for Detection of Cancer Cell Invasion

Cell invasion was evaluated using Transwell chamber inserts equipped with polyvinylpyrrolidone-free polycarbonate membranes (8 μm pore size; BD Biosciences, Franklin Lakes, NJ, USA). For invasion analysis, the upper surface of each membrane was coated with 15 μg of Matrigel^®^ (Corning^®^ Basement Membrane Matrix, Cat. No. 356234, Corning Life Sciences, Syracuse, NY, USA) and allowed to solidify at 37 °C prior to cell seeding. A549 cells were suspended in serum-reduced DMEM (0.5% FBS) and seeded into the upper chamber at a density of 8 × 10^4^ cells per insert. The lower chamber was filled with DMEM containing 10% FBS to serve as a chemoattractant. To assess the pro-invasive effect of inflammatory stimulation, cells were exposed to standardized THP-1-conditioned medium (THP-1-CS), enriched in IL-6 (234.88 ± 7.36 pg/mL) and IL-1β (124.55 ± 10.25 pg/mL) with a volume of 1 mL, for 24 h. For cytokine neutralization experiments, anti-IL-6 (1:1000) and anti-IL-1β antibodies (1:1000) were added to the culture system to determine the specific contribution of these cytokines to invasion.

To evaluate the inhibitory effects of CKAB-P1 and C3G, A549 cells were pretreated with CKAB-P1 or C3G for 4 h prior to THP-1-CS stimulation. Cells were then incubated for an additional 24 h under inflammatory conditions. Membranes were rinsed with distilled water to remove excess stain. Invaded cells were visualized under a phase-contrast microscope, and representative images were captured. Quantification was performed using the “Threshold” function in ImageJ software (version 1.410). Threshold parameters were adjusted consistently across all groups to distinguish stained cells from background. For each insert, at least three randomly selected microscopic fields were analyzed, and the average value was used for statistical comparison.

### 2.10. MMP-2 and MMP-9 Activity by Gelatin Zymography

Gelatin, a denatured form of collagen, was incorporated into polyacrylamide gels as a substrate to detect the gelatinolytic activity of MMP-2 (gelatinase A) and MMP-9 (gelatinase B). The cells were seeded in 6-well plates at a density of 2 × 10^5^ cells per well and incubated overnight. Cells were pretreated with CKAB-P1 or C3G for 4 h prior to stimulation with standardized THP-1-conditioned medium (THP-1-CS), enriched in IL-6 (234.88 ± 7.36 pg/mL) and IL-1β (124.55 ± 10.25 pg/mL) with a volume of 2 mL, for an additional 24 h. Equal amounts of protein from conditioned media were subjected to electrophoresis on 10% SDS-polyacrylamide gels containing 0.1 mg/mL gelatin under non-reducing conditions. The gels were stained with 0.1% Coomassie Brilliant Blue R-250 (Thermo Fisher Scientific, Rockford, IL, USA). Clear bands of MMP-9 (92 kDa) and MMP-2 (72 kDa) were visualized and quantified using ImageJ software (version 1.410).

### 2.11. Western Blot Analysis

Protein expression levels of invasion-associated proteins and JAK/STAT signaling components were analyzed by Western blotting. The cells (3 × 10^5^ cells per well) were seeded in 6-well plates and pretreated with CKAB-P1 or C3G for 4 h prior to stimulation with standardized THP-1-conditioned medium (THP-1-CS), enriched in IL-6 (234.88 ± 7.36 pg/mL) and IL-1β (124.55 ± 10.25 pg/mL) with a volume of 2 mL, for 24 h.

Following treatment, cells were harvested and lysed in RIPA buffer supplemented with protease inhibitor cocktail (Thermo Fisher Scientific, Rockford, IL, USA). Equal amounts of total protein (30 μg per lane) were separated by 12% SDS–PAGE and transferred onto nitrocellulose membranes. The membranes were incubated overnight at 4 °C with primary antibodies against uPA, uPAR, MT1-MMP, phosphorylated JAK1 (*p*-JAK1), total JAK1, phosphorylated STAT3 (*p*-STAT3), and total STAT3, all at 1:2000 dilution, and the antibodies were obtained from Cell Signaling Technology, Danvers, MA, USA. The membranes were incubated with HRP-conjugated secondary antibodies (Cell Signaling Technology, Danvers, MA, USA) at a 1:10,000 ratio of anti-mouse or anti-rabbit IgG for 2 h. The bands were visualized using enhanced chemiluminescence reagents (Cytiva, Marlborough, MA, USA) and captured with the iBright™ CL-1500 imaging system (Thermo Fisher Scientific, Waltham, MA, USA). To verify equal protein loading, membranes were stripped and reprobed with anti-β-actin antibody (Sigma-Aldrich, St. Louis, MO, USA) at 1:10,000 ratio. Band intensities were quantified using ImageJ software (version 1.410).

### 2.12. Statistical Analysis

All data are presented as mean ± standard deviation (SD). The one-way analysis of variance (ANOVA) followed by Dunnett’s test or Tukey’s test was used for statistical analysis using GraphPad Prism version 8.0 (GraphPad Software, San Diego, CA, USA). Statistical significance was considered at *p* < 0.05 (*), *p* < 0.01 (**), and *p* < 0.001 (***).

## 3. Results

### 3.1. Phytochemical Profile of C3G-Rich Fractions

Two fractions derived from Kum Akha 1 CMU black rice were obtained through solvent-partition extraction and designated CKAB-P1 (butanol fraction) and CKAB-P2 (aqueous fraction). The % yields of CKAB-P1 and CKAB-P2 were 3.64 ± 1.06% and 7.08 ± 0.52% (*w*/*w*), respectively. The phytochemical compositions of both fractions are summarized in Table 1. CKAB-P1 contained significantly greater total phenolic content (TPC), total flavonoid content (TFC), and total anthocyanin content (TAC) compared with CKAB-P2 (* *p* < 0.05). Specifically, CKAB-P1 exhibited 165.53 ± 10.59 mg GAE/g extract of total phenolics and 134.05 ± 5.27 mg CE/g extract of total flavonoids, which were markedly greater than those quantified in CKAB-P2.

**Figure 1:**
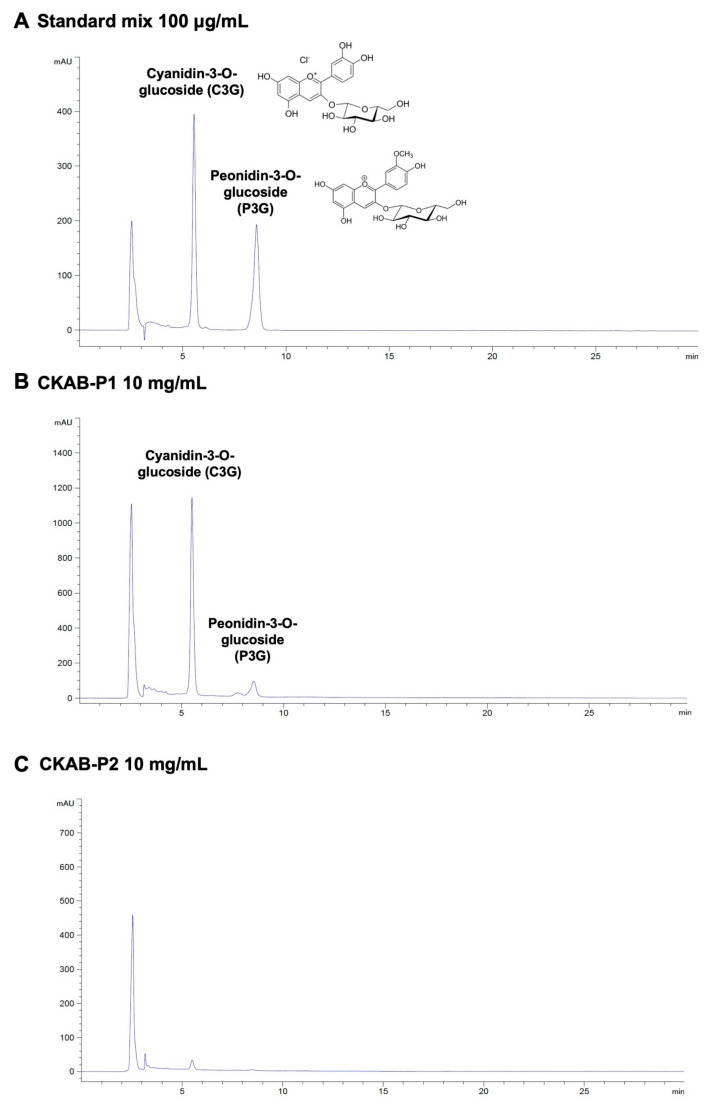
HPLC profiles of anthocyanins in Kum Akha 1 CMU black rice germ and bran fractions. (**A**) Chromatogram of authentic anthocyanin standards, cyanidin-3-O-glucoside (C3G) and peonidin-3-O-glucoside (P3G), each at 100 μg/mL, showing their respective retention times. (**B**) Chromatogram of CKAB-P1 at 10 mg/mL. (**C**) Chromatogram of CKAB-P2 at 10 mg/mL.

Regarding anthocyanins, CKAB-P1 demonstrated a significantly higher total anthocyanin content (106.62 ± 3.54 mg/g extract) compared with CKAB-P2 (15.70 ± 1.67 mg/g extract; *p* < 0.05), indicating effective enrichment of anthocyanins in the butanol fraction. To further characterize anthocyanin composition, HPLC analysis was performed (Figure 1). Representative chromatograms of anthocyanin standards confirmed the retention times of C3G and P3G. Quantitative analysis revealed that CKAB-P1 contained 59.42 ± 2.54 mg/g extract of C3G and 10.60 ± 0.37 mg/g extract of P3G. In contrast, neither C3G nor P3G was detected in CKAB-P2. Collectively, these findings confirm that CKAB-P1 represents an anthocyanin-enriched fraction, with C3G as the predominant anthocyanin compound.

### 3.2. Antioxidant Properties of C3G-Rich Fractions from Black Rice

The inhibitory concentration at 50% values (IC_50_) of CKAB-P1 and CKAB-P2 are summarized in Table 2. For the DPPH assay, vitamin E was used as a positive control and exhibited an IC_50_ value of 29.04 ± 1.32 μg/mL. In the ABTS assay, Trolox served as the reference antioxidant with an IC_50_ value of 2.40 ± 0.27 μg/mL. Among the two fractions, CKAB-P1 demonstrated significantly stronger radical-scavenging activity than CKAB-P2, as indicated by its lower IC_50_ values in both DPPH and ABTS assays (* *p* < 0.05). CKAB-P1 exhibited IC_50_ values of 31.35 ± 2.30 μg/mL (DPPH) and 16.43 ± 0.59 μg/mL (ABTS), both significantly lower than those of CKAB-P2. Based on its enriched C3G content and superior antioxidant capacity, CKAB-P1 was selected for subsequent biological investigations alongside the C3G compound.

### 3.3. Effects of CKAB-P1 and C3G on A549 Lung Cancer Cell Cytotoxicity

**Figure 2:**
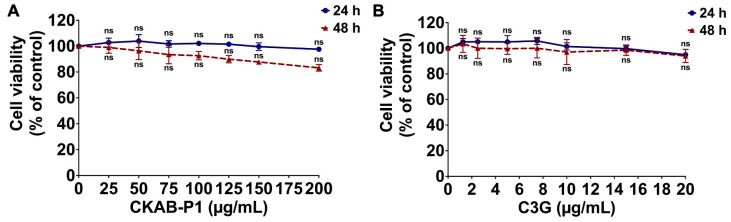
Effects of CKAB-P1 (**A**) and C3G (**B**) on A549 cell viability using the MTT assay. Data are given as mean ± standard deviation (SD). ns = No statistically significant differences vs. untreated controls (*n* = 3).

Prior to evaluating the inhibitory effects of CKAB-P1 and C3G on inflammation-driven cancer progression, cytotoxicity was assessed using the MTT assay to determine non-toxic concentration ranges in A549 cells. Cell viability was expressed as a percentage relative to untreated controls following exposure to increasing concentrations of CKAB-P1 (0–200 μg/mL) or C3G (0–20 μg/mL) for 24 and 48 h. As shown in Figure 2, neither CKAB-P1 nor C3G significantly reduced A549 cell viability within the tested concentration ranges at either time point. The calculated IC_50_ values for CKAB-P1 were greater than 200 μg/mL at both 24 and 48 h. Similarly, the IC_50_ values for C3G exceeded 20 μg/mL under the same experimental conditions.

### 3.4. Establishment of an Inflammation-Driven Tumor Microenvironment Using Conditioned Medium from LPS-Stimulated THP-1 Macrophages (THP-1-CS)

**Figure 3:**
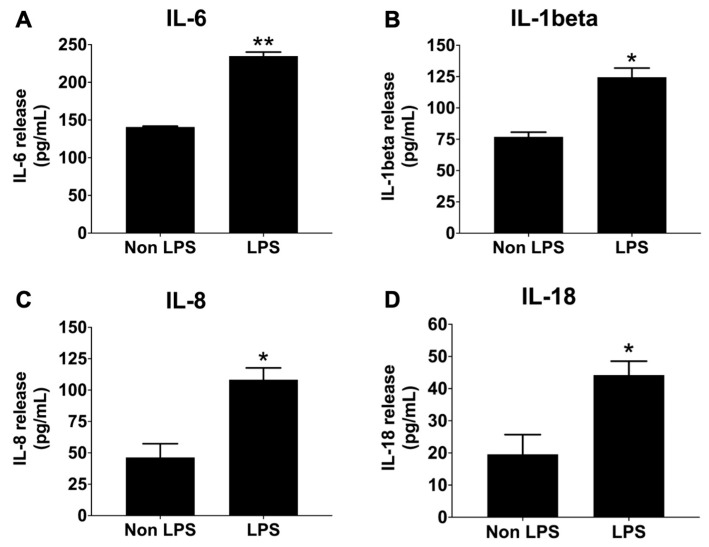
Pro-inflammatory cytokine concentrations in conditioned medium from LPS-stimulated THP-1 macrophages (THP-1-CS). Levels of IL-6 (**A**), IL-1β (**B**), IL-8 (**C**), and IL-18 (**D**) in culture supernatants were quantified by ELISA following stimulation with 1 μg/mL LPS for 24 h. Data are given as mean ± standard deviation (SD). * *p* < 0.05, ** *p* < 0.01 vs. non-LPS-treated THP-1 control (*n* = 3).

To model an inflammation-driven tumor microenvironment in vitro, conditioned medium derived from lipopolysaccharide (LPS)-stimulated THP-1 macrophages was generated and characterized prior to its application in A549 cells. The set for the experiment was modified from previously described protocols and applied in our THP-1 and A549 models [34,35]. Following LPS stimulation, culture supernatants were collected, and the concentrations of IL-6, IL-8, IL-1β, and IL-18 cytokines were quantified by ELISA. Cytokine levels were compared with those in supernatants from non-stimulated THP-1 macrophages. As shown in Figure 3, LPS exposure significantly increased the secretion of IL-6, IL-8, IL-1β, and IL-18 compared with the non-LPS control group (*p* < 0.05). Among the detected cytokines, IL-6 exhibited the highest concentration (234.88 ± 7.36 pg/mL), followed by IL-1β (124.55 ± 10.25 pg/mL), IL-8 (108.33 ± 16.17 pg/mL), and IL-18 (44.23 ± 7.42 pg/mL).

These findings confirm successful induction of an inflammatory phenotype in THP-1 macrophages. The resulting conditioned medium, designated THP-1-CS, was subsequently used to simulate an inflammation-driven tumor microenvironment in A549 cells. Cytokine concentrations were standardized prior to application in downstream migration and invasion assays. The volume of conditioned medium applied to A549 cells was adjusted to ensure consistent cytokine exposure across experiments.

### 3.5. Effects of CKAB-P1 and C3G on THP-1-CS-Induced A549 Cell Migration and Invasion

**Figure 4:**
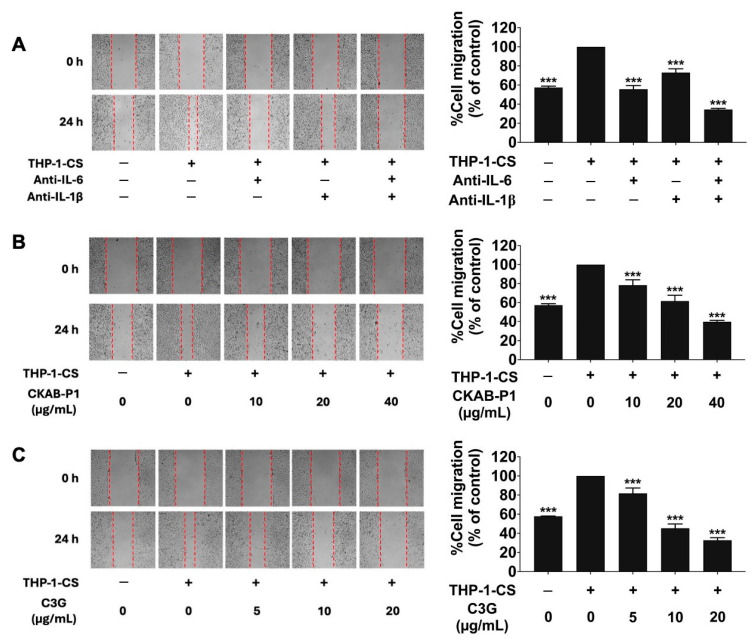
Effects of THP-1-conditioned medium (THP-1-CS) and cytokine neutralization on A549 cell migration. Migration of A549 cells following exposure to THP-1-CS with or without neutralizing antibodies against IL-6 and IL-1β (**A**). Inhibitory effects of CKAB-P1 (**B**) and C3G (**C**) on THP-1-CS-induced A549 cell migration assessed by wound-healing assay. Migration in the THP-1-CS-treated group was set as 100%. Data are given as mean ± standard deviation (SD). *** *p* < 0.001 versus THP-1-CS-treated group (*n* = 3).

In the wound-healing assay as shown in Figure 4, exposure to THP-1-CS significantly increased the migratory capacity of A549 cells compared with non-induced controls (Figure 4A). Neutralization experiments revealed that treatment with anti-IL-6 or anti-IL-1β antibodies partially reduced THP-1-CS-induced migration, whereas combined antibody treatment almost completely abolished the enhanced migratory response. The inhibitory effects of CKAB-P1 and C3G were subsequently examined. Non-cytotoxic concentrations were selected to minimize potential proliferation-related confounding effects.

**Figure 5:**
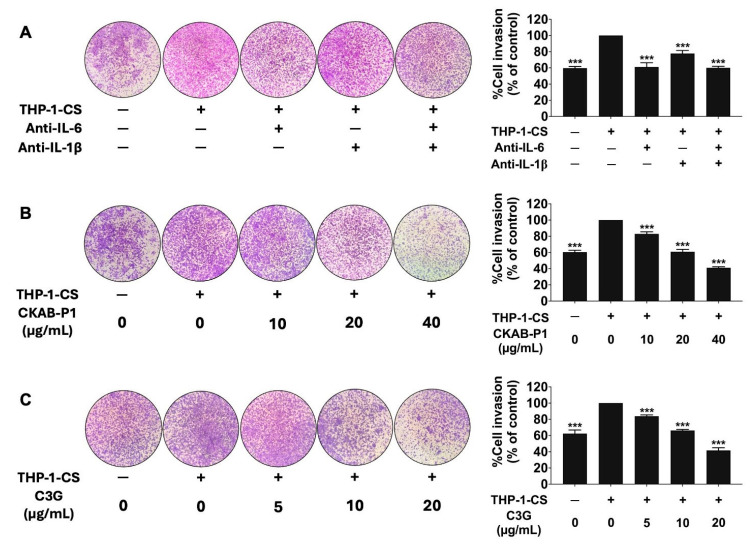
Effects of THP-1-conditioned medium (THP-1-CS) and cytokine neutralization on A549 cell invasion. Invasive capacity of A549 cells following exposure to THP-1-CS with or without neutralizing antibodies against IL-6 and IL-1β (**A**). Inhibitory effects of CKAB-P1 (**B**) and C3G (**C**) on THP-1-CS-induced A549 cell invasion assessed by Transwell assay. Invasion in the THP-1-CS-treated group was set as 100%. Data are given as mean ± standard deviation (SD). *** *p* < 0.001 versus the THP-1-CS-treated group (*n* = 3).

As shown in Figure 4B,C, both CKAB-P1 and C3G significantly attenuated THP-1-CS-induced A549 cell migration in a dose-dependent manner (Figure 4B,C; *p* < 0.001). Consistent results were obtained in the Transwell invasion assay, as shown in Figure 5. THP-1-CS exposure markedly increased the invasive capacity of A549 cells relative to non-induced cells (Figure 5A). Neutralization of IL-6 or IL-1β reduced invasion, and simultaneous blockade of both cytokines nearly completely reversed the THP-1-CS-mediated invasive effect. Treatment with CKAB-P1 or C3G significantly attenuated THP-1-CS-induced invasion in a dose-dependent manner (Figure 5B,C; *p* < 0.001). Collectively, these findings demonstrate that THP-1-CS enhances A549 cell migration and invasion through cytokine-mediated mechanisms involving IL-6 and IL-1β. Importantly, both CKAB-P1 and C3G effectively counteracted these inflammation-driven metastatic phenotypes.

### 3.6. Effects of CKAB-P1 and C3G on MMP-2 and MMP-9 Activities and Invasive Protein Expression in THP-1-CS-Induced A549 Cell Invasion

**Figure 6:**
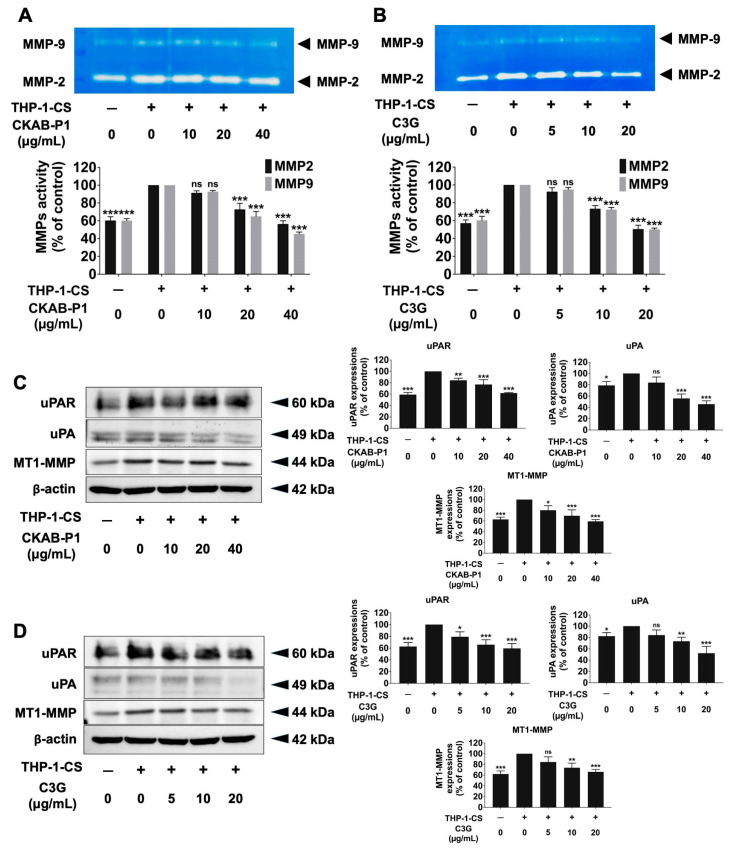
Effects of CKAB-P1 and C3G on MMP activity and invasion-related protein expression in THP-1-CS-induced A549 cells. Inhibitory effects of (**A**) CKAB-P1 and (**B**) C3G on MMP-9 (92 kDa) and MMP-2 (72 kDa) activity in THP-1-CS-induced A549 cells. Inhibitory effects of (**C**) CKAB-P1 and (**D**) C3G on uPAR (60 kDa), uPA (49 kDa), and MT1-MMP (44 kDa) protein expression in THP-1-CS-induced A549 cells. The quantitative densitometric analysis of u-PAR, u-PA, and MT1-MMP, normalized to β-actin (42 kDa) as the loading control. Data are given as mean ± standard deviation (SD). * *p* < 0.05, ** *p* < 0.01, and *** *p* < 0.001 versus the THP-1-CS-treated group. No statistically significant differences were observed compared with untreated controls (ns) (*n* = 3).

The gelatinolytic activity corresponding to MMP-9 (92 kDa) and MMP-2 (72 kDa) was detected as clear bands on polyacrylamide gels and is shown in Figure 6. Exposure to THP-1-CS increased the activity of both MMP-9 and MMP-2 compared with non-induced controls. Treatment with CKAB-P1 or C3G significantly reduced the activity of MMP-9 and MMP-2 in a dose-dependent manner (Figure 6A,B; *p* < 0.001), consistent with the observed attenuation of invasive capacity. To further substantiate these findings, the expression levels of key invasion-associated proteins were assessed by Western blot analysis. CKAB-P1 and C3G markedly downregulated the protein expression of uPA, uPAR, and MT1-MMP in THP-1-CS-induced A549 cells in a dose-dependent manner (Figure 6C,D; *p* < 0.001). Collectively, these results demonstrate that CKAB-P1 and C3G suppress inflammation-enhanced invasive phenotypes by reducing MMP activity and downregulating invasion-related protein expression in A549 lung cancer cells.

### 3.7. Effects of CKAB-P1 and C3G on JAK1/STAT3 Signaling in THP-1-CS-Induced A549 Cells

**Figure 7:**
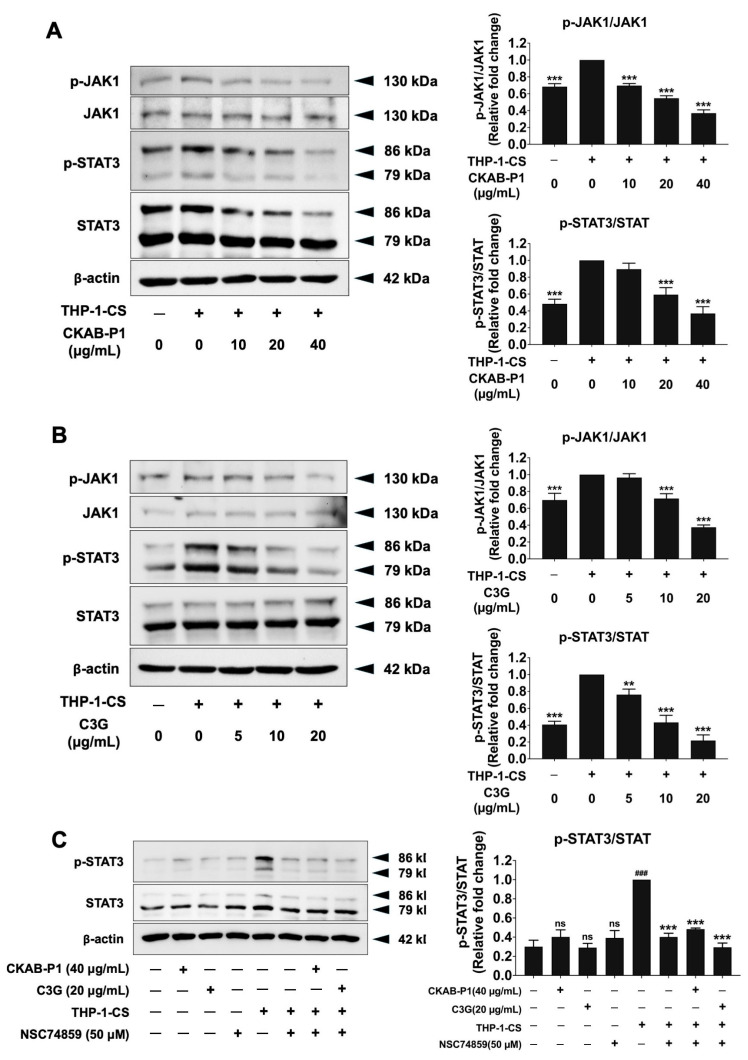
Effects of CKAB-P1 and C3G on JAK1/STAT3 signaling in THP-1-CS-induced A549 cells. (**A**,**B**) The inhibitory effects of CKAB-P1 (**A**) and C3G (**B**) on the phosphorylation of the JAK1 and STAT3 proteins in THP-1-CS-induced A549 cells are displayed in Western blot and band density measurements. (**C**) The effect of STAT3 inhibitor (NSC 74859) on phosphorylation of STAT3 proteins in THP-1-CS-stimulated A549 cells. Phosphorylated protein levels are normalized to their respective total JAK1 or STAT3 levels and expressed relative to the THP-1-CS-treated group, which was set as 1.0. Data are given as mean ± standard deviation (SD) from three independent experiments (*n* = 3). ** *p* < 0.01, *** *p* < 0.001 versus the THP-1-CS-treated group; ^###^ *p* < 0.001 versus the unstimulated control group; ns, non-statistically significant differences compared with the unstimulated control group.

The anti-migratory and anti-invasive effects of CKAB-P1 and C3G via modulation of the JAK1/STAT3 pathway were examined by Western blot analysis. Exposure to THP-1-conditioned medium (THP-1-CS) significantly increased the phosphorylation levels of JAK1 and STAT3 in A549 cells compared with non-induced controls (*p* < 0.001), indicating activation of the JAK1/STAT3 pathway under inflammatory conditions (Figure 7). Treatment with CKAB-P1 or C3G markedly reduced THP-1-CS-induced phosphorylation of JAK1 and STAT3 in a dose-dependent manner (Figure 7A,B; *p* < 0.001), while total JAK1 and STAT3 protein levels remained unchanged. To confirm whether THP-1-CS stimulation activates the STAT3 signaling pathway in A549 cells, the cells were treated with the STAT3 inhibitor NSC74859. As shown in Figure 7C, in the absence of THP-1-CS stimulation, the levels of phosphorylated STAT3 were unchanged among CKAB-P1, C3G, and NSC74859 treatments when compared with the unstimulated control group (*p* > 0.05). When stimulated with THP-1-CS alone, A549 cells exhibited significantly increased levels of phosphorylated STAT3 (*p*-STAT3) when compared with the unstimulated control group (*p* < 0.001). Furthermore, treatment with the STAT3 inhibitor significantly reduced *p*-STAT3 levels in THP-1-CS-stimulated A549 cells (*p* < 0.001). These findings indicate that THP-1-CS activates STAT3 signaling in A549 cells, which potentially leads to A549 cell migration and invasion through activation of the STAT3 signaling pathway. Collectively, these findings suggest that CKAB-P1 and C3G attenuate inflammation-enhanced metastatic phenotypes in A549 cells, at least in part, through suppression of JAK1/STAT3 signaling activation.

## 4. Discussion

Anthocyanins are major bioactive constituents of pigmented rice varieties, particularly concentrated in the germ and bran layers [18,26]. Among them, C3G is the predominant anthocyanin identified in black rice [17,36]. Beyond their antioxidant properties, anthocyanins have been increasingly recognized for anti-inflammatory and anti-metastatic activities across multiple cancer models [37,38,39]. Previous studies have demonstrated that anthocyanin-rich black rice extracts suppress inflammatory cytokine production in macrophages through modulation of NF-κB/MAPK signaling and inflammasome-related pathways [22,40]. Additionally, C3G has been reported to attenuate inflammatory mediator release and oxidative stress in respiratory inflammation in both cell culture and animal models, primarily through regulation of redox balance and inflammation-associated signaling pathways [41].

One important factor underlying the bioactivity of C3G is its antioxidant mechanism. C3G and other polyphenols may act either as direct antioxidants or indirectly by modulating endogenous antioxidant defense systems and regulating the expression of antioxidant proteins. Notably, polyphenols may exhibit both antioxidant and pro-oxidant activities depending on concentration and cellular context, a phenomenon commonly observed for natural antioxidants [42,43]. It should also be noted that CKAB-P1 represents a complex phytochemical mixture, and although C3G is the predominant anthocyanin, potential synergistic or additive effects of other co-extracted constituents cannot be excluded. Further detailed chemical profiling and bioactivity-guided fractionation would be valuable to identify additional active components and clarify their contributions.

While the anti-inflammatory effects of black rice anthocyanins are well established, their role in modulating inflammation-enhanced tumor progression and cytokine-driven JAK/STAT signaling remains unclear. Herein, we provided the scientific support that the C3G-enriched fraction from pigmented black rice suppresses macrophage-derived IL-6/IL-1β-mediated A549 lung cancer cell progression and inhibits JAK1/STAT3 signaling activation. This potential mechanistic link between dietary anthocyanins and cytokine-dependent A549 lung cancer progression could offer novel insight into how functional foods may modulate one of the hallmarks of cancer, tumor-promoting inflammation [44].

The present study demonstrated that THP-1-derived conditioned medium significantly elevated IL-6 and IL-1β levels and enhanced A549 cell migration and invasion. This observation is consistent with established macrophage polarization models in which LPS stimulation induces a pro-inflammatory (M1-like) phenotype associated with increased production of IL-6 and IL-1β [45,46]. Neutralization of these cytokines markedly reduced metastatic phenotypes, supporting their pivotal role in tumor progression [47,48]. Moreover, a previous study also found that various ratios of THP-1-derived conditioned medium in the presence of LPS had a slight influence on the growth ability of A549 cells [34]. In the present study, to minimize the potential influence of cell proliferation on the interpretation of migration and invasion results, all experiments were conducted under reduced-serum conditions (0.5% FBS in DMEM). Under these conditions, the availability of growth factors is limited, thereby reducing proliferation-related interference. In addition, cells were exposed to CKAB-P1 and C3G for no longer than 24 h, as this study was not designed to evaluate proliferation effects. Importantly, cell viability assays (MTTs) confirmed that CKAB-P1 and C3G did not significantly affect A549 cell viability at the concentrations used in migration and invasion experiments, indicating that the observed inhibitory effects are unlikely to be attributed to reduced cell proliferation. Nevertheless, it should be noted that wound-healing assays may be partially influenced by cell proliferation, and the use of proliferation inhibitors such as mitomycin C could further strengthen migration-specific interpretation in future studies.

NSCLC, particularly adenocarcinoma, frequently exhibits aberrant activation of oncogenic signaling, including KRAS, PI3K/Akt, and JAK/STAT cascades [34,49,50]. Although direct targeting of mutant KRAS remains challenging, modulation of downstream inflammatory signaling networks has emerged as a complementary therapeutic strategy [51]. The poor prognosis and enhanced metastatic potential in NSCLC are partly related to sustained activation of IL-6/STAT3 signaling [52]. Therefore, dietary bioactive compounds capable of attenuating cytokine-driven STAT3 activation may represent supportive strategies for limiting inflammation-associated tumor progression. Consistent with this concept, several diet-derived bioactive compounds have been reported to suppress inflammation-associated STAT3 activation and metastatic phenotypes in cancer models. For instance, wogonin inhibited A549 cell migration in inflammation-induced cancer progression through modulation of the IL-6/STAT3 axis [34]. More broadly, the IL-6/JAK/STAT3 cascade is recognized as a central regulator of tumor progression and metastasis within the inflammatory tumor microenvironment [52,53]. These findings indicate that interference with cytokine-linked STAT3 activation represents a common anti-metastatic mechanism among polyphenol-type compounds. Importantly, our study extends these observations by demonstrating that a naturally derived C3G-rich fraction from pigmented black rice attenuates macrophage-driven inflammatory signaling and its downstream invasive machinery within a cytokine-standardized microenvironment model.

IL-6-mediated STAT3 activation is widely implicated in cancer cell survival, invasion, and immune evasion [40,41], while IL-1β contributes to tumor-promoting inflammation and extracellular matrix remodeling [42]. Upon activation, phosphorylated STAT3 undergoes dimerization and translocates into the nucleus to regulate transcription of genes associated with cell survival, invasion, and metastasis, including matrix metalloproteinases and components of the urokinase system [54,55]. Our findings indicated that THP-1-CS induces JAK1 and STAT3 protein phosphorylation in A549 cells, thereby promoting the expression of downstream invasion-associated mediators. Importantly, CKAB-P1 and C3G significantly reduced THP-1-CS-induced phosphorylation of JAK1 and STAT3 without altering total protein expression, indicating selective inhibition of pathway activation. Since STAT3 regulates transcription of metastasis-associated genes, including MMP-2, MMP-9, and components of the urokinase system [56,57], suppression of this signaling axis could provide an explanation for the observed reductions in gelatinase activity and invasive-protein expression. Although nuclear translocation of STAT3 was not directly evaluated in this study, inhibition of STAT3 phosphorylation is widely recognized to impair its nuclear localization and transcriptional activity, thereby attenuating downstream pro-metastatic signaling [57]. These findings support a mechanistic link between anthocyanin-rich fractions and cytokine-driven A549 cancer signaling pathways at the level of phosphorylation modulation. It should be noted that the present study does not identify the direct molecular targets of CKAB-P1 or C3G, such as specific receptors or upstream kinases. Therefore, further studies are required to elucidate the precise molecular interactions and determine whether they coordinate with the downregulation of MMP activity and uPA/uPAR/MT1-MMP expression.

Despite these favorable findings, it is worth mentioning that this study was conducted solely on a single A549 cell line, which may not completely reflect the molecular heterogeneity of lung cancer. With regard to our in vitro investigation of the cytokine-induced cancer progression model, we selected A549 cells as our cell culture model because this cancer cell line was commonly used in the experiments of previous studies that focused on cancer studies and inflammation-related lung cancer cell tumorigenesis [34,58,59,60]. Accordingly, these aforementioned studies used the same A549 cells to represent the cancer cells that were induced by cytokines or inflammatory mediators to enhance their aggressiveness. Nevertheless, further validation in additional NSCLC models, including KRAS-mutant and EGFR-mutant cell lines, is warranted to better capture the molecular heterogeneity of lung cancer and strengthen translational relevance [61]. Moreover, although the LPS-stimulated THP-1 macrophage-conditioned medium model provides a controllable inflammatory system, it does not fully recapitulate the complexity of tumor-associated macrophages and stromal interactions in vivo. This controlled system, however, enables mechanistic investigation of cytokine-driven inflammation-induced cancer progression. Future studies incorporating co-culture systems or in vivo models would provide more physiologically relevant validation.

Lastly, the bioavailability and metabolic stability of C3G following dietary intake remain important considerations, as anthocyanins undergo extensive metabolism in the gastrointestinal tract and circulation [62,63,64]. Regarding the pharmacokinetics in animal and human studies, a shorter half-life of C3G (0.7–1.8 h) was reported following a 500 mg/kg oral dose in a mouse model [65]. A study on humans found that after a 500 mg oral dose, C3G metabolites had half-lives ranging between 12.44 and 51.62 h, suggesting the prolonged presence of C3G in blood circulation [66]. Additionally, CKAB-P1 represents a complex phytochemical mixture, and although C3G is the predominant anthocyanin, potential synergistic or additive effects of other co-extracted constituents cannot be excluded. Further comprehensive chemical profiling and bioactivity-guided fractionation would be valuable to identify additional active components and clarify their contributions. Future in vivo studies are warranted to evaluate pharmacokinetics, tissue distribution, effective dosing, safety profiles, and potential synergistic effects with existing therapies. Further exploration of upstream receptor interactions and crosstalk with other oncogenic pathways, including NF-κB and PI3K/Akt signaling, would also provide deeper mechanistic insight.

## 5. Conclusions

In summary, a cyanidin-3-O-glucoside (C3G)-rich fraction derived from Kum Akha pigmented black rice (CKAB-P1) effectively attenuated IL-6/IL-1β inflammation-driven migration and invasion of A549 lung cancer cells. These findings indicate that modulation of the IL-6/IL-1β-induced A549 lung cancer migration and invasion represents a key mechanism underlying the inhibitory effects of anthocyanin-rich black rice. Using a cytokine-enriched macrophage-conditioned medium model, we demonstrated that CKAB-P1 and C3G attenuated A549 cell migration and invasion capabilities through inhibition of MMP-2 and MMP-9 activity, downregulation of uPA/uPAR/MT1-MMP expression, and attenuation of JAK1/STAT3 phosphorylation. Collectively, this study provides the potential role of dietary anthocyanins in targeting tumor-promoting inflammation associated with A549 lung cancer progression, although further studies are required to confirm direct molecular targets and in vivo applicability.”

## Figures and Tables

**Table 1:** Phytochemical composition of Kum Akha 1 CMU black rice fractions (CKAB-P1 and CKAB-P2).

Assay	CKAB-P1	CKAB-P2
TPC (mg GAE/g extract)	165.53 ± 10.59 *	72.62 ± 3.37
TFC (mg CE/g extract)	134.05 ± 5.27 *	80.76 ± 7.84
TAC (mg/g extract)	106.62 ± 3.54 *	15.70 ± 1.67
C3G (mg/g extract)	59.42 ± 2.54	n.d.
P3G (mg/g extract)	10.60 ± 0.37	n.d.

Data are given as mean ± standard deviation (SD). TPC = Total phenolic content. TFC = otal flavonoid content. TAC = Total anthocyanin content. n.d. = Not detected. * *p* < 0.05 indicates being statistically significant vs. CKAB-P2.

**Table 2:** Antioxidant activities of Kum Akha 1 CMU black rice fractions (CKAB-P1 and CKAB-P2) evaluated by DPPH and ABTS assays.

Extract or Compound	IC_50_ (μg/mL)	
	DPPH Assay	ABTS Assay
CKAB-P1	31.35 ± 2.30 *	16.43 ± 0.59 *
CKAB-P2	201.28 ± 28.23	60.35 ± 0.42
Vitamin E	29.04 ± 1.32	-
Trolox	-	2.40 ± 0.27

Data are given as mean ± standard deviation (SD). Vitamin E and Trolox were used as positive controls for the DPPH and ABTS assays, respectively. * *p* < 0.05 indicates being statistically significant vs. CKAB-P2.

## Data Availability

Data are contained within the article.

## Supplementary Materials

The following supporting information can be downloaded at https://www.mdpi.com/article/10.3390/nu18081198/s1: Figure S1: Representative chemical structures of major phenolic compounds found in black rice.

## Abbreviations

The following abbreviations are used in this manuscript:

C3G	Cyanidin-3-O-glucoside
CKAB-P1	Cyanidin-3-O-glucoside-rich fraction from Kum Akha pigmented black rice
THP-1-CS	Conditioned medium from LPS-exposed THP-1 macrophages
IL-6	Interleukin-6
IL-1β	Interleukin-1beta
MMP-2	Matrix metalloproteinase-2
MMP-9	Matrix metalloproteinase-9
uPA	Urokinase-type plasminogen activator
uPAR	Urokinase-type plasminogen activator receptor
MT1-MMP	Membrane type 1-matrix metalloproteinase
JAK1	Janus kinase 1
STAT3	Signal transducer and activator of transcription 3
NSCLC	Non-small cell lung cancer
TPC	Total phenolic content
TFC	Total flavonoid content
TAC	Total anthocyanin content
GAE	Gallic acid equivalent
CE	Catechin equivalent
HPLC	High-performance liquid chromatography
P3G	Peonidin-3-O-glucoside
mg	Milligram
g	gram
ABTS	2,2′-azino-bis (3-ethylbenzothiazoline-6-sulfonic acid)
DPPH	2,2-diphenyl-1-picrylhydrazyl
IC_50_	Inhibitory concentration at 50%
MTT	3-(4,5-Dimethylthiazol-2-yl)-2,5-Diphenyltetrazolium Bromide
LPS	Lipopolysaccharide
PMA	Phorbol 12-myristate 13-acetate
