# Supplementary material for: Cyanidin-3-O-Glucoside-Rich Black Rice Fraction Attenuates IL-1β/IL-6-Driven A549 Lung Cancer Cell Migration and Invasion and Modulates JAK1/STAT3 Signaling
Source: Nutrients. 2026 Apr 10;18(8):1198. doi: 10.3390/nu18081198 (PMC13118757; doi:10.3390/nu18081198)
Supplement: Supplementary file 1 [file nutrients-18-01198-s001.zip › nutrients-4218842-supplementary.pdf]

Supplementary Data

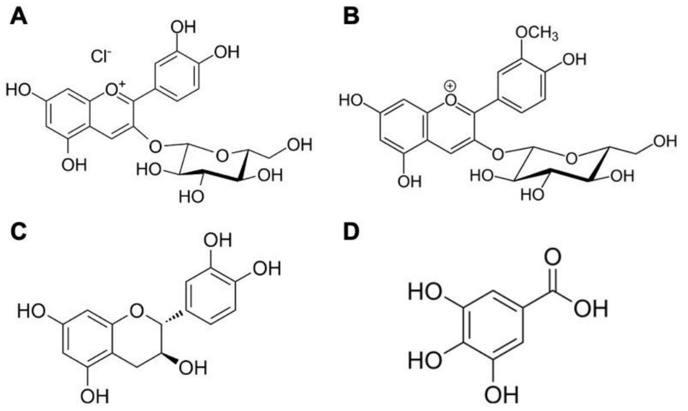

Figure S1. Representative chemical structures of major phenolic compounds found in black rice. (A) Cyanidin-3-O-glucoside (C3G); (B) Peonidin-3-O-glucoside (P3G); (C) Catechin; (D) Gallic acid. These compounds represent major phenolic subclasses, including anthocyanins, flavonoids, and phenolic acids commonly present in pigmented rice.

Panel A — C3G
Label: (A) Cyanidin-3-O-glucoside (C3G)

- Molecular formula: $\mathbf{C_{21}H_{21}O_{11}^{+}}$
- Molecular weight: **449.38 g/mol**
- CAS: **7084-24-4**

Panel B — P3G
Label: (B) Peonidin-3-O-glucoside (P3G)

- Molecular formula: $\mathbf{C_{22}H_{23}O_{11}^{+}}$
- Molecular weight: **463.41 g/mol**
- CAS: **27200-12-0**

Panel C — Catechin
Label: (C) Catechin (CE)

- Molecular formula: $\mathbf{C_{15}H_{14}O_{6}}$
- Molecular weight: **290.27 g/mol**
- CAS: **154-23-4**

Panel D — Gallic acid
Label: (D) Gallic acid (GA)

- Molecular formula: $\mathbf{C_{7}H_{6}O_{5}}$
- Molecular weight: **170.12 g/mol**
- CAS: **149-91-7**
